# Content of Two Major Steroidal Glycoalkaloids in Tomato (*Solanum lycopersicum* cv. Micro-Tom) Mutant Lines at Different Ripening Stages

**DOI:** 10.3390/plants11212895

**Published:** 2022-10-28

**Authors:** Trung Huy Ngo, Jisu Park, Yeong Deuk Jo, Chang Hyun Jin, Chan-Hun Jung, Bomi Nam, Ah-Reum Han, Joo-Won Nam

**Affiliations:** 1 College of Pharmacy, Yeungnam University, Gyeongsan-si 38541, Gyeongsangbuk-do, Korea; 2 Advanced Radiation Technology Institute, Korea Atomic Energy Research Institute, Jeongeup-si 56212, Jeollabuk-do, Korea; 3 College of Agriculture and Life Sciences, Chungnam National University, Daejeon 34134, Chungcheongnam-do, Korea; 4 Jeonju AgroBio-Materials Institute, Jeonju-si 54810, Jeollabuk-do, Korea; 5 Institute of Natural Cosmetic Industry for Namwon, Namwon-si 55801, Jeollabuk-do, Korea

**Keywords:** *Solanum lycopersicum*, radiation breeding, esculeoside A, tomatine, UPLC-ELSD

## Abstract

Esculeoside A and tomatine are two major steroidal alkaloids in tomato fruit (*Solanum lycopersicum*) that exhibit anti-inflammatory, anticancer, and anti-hyperlipidemia activities. Tomatine contained in immature tomato fruit is converted to esculeoside A as the fruit matures. To develop new tomato varieties based on the content analysis of functional secondary metabolites, 184 mutant lines were generated from the original cultivar (*S. lycopersicum* cv. Micro-Tom) by radiation breeding. Ultra-performance liquid chromatography coupled with evaporative light scattering detector was used to identify the mutant lines with good traits by analyzing tomatine and esculeoside A content. Compared with the original cultivar, candidates for highly functional cultivars with high esculeoside A content were identified in the mature fruit of the mutant lines. The mutant lines with low and high tomatine content at an immature stage were selected as edible cultivars due to toxicity reduction and as a source of tomatine with various pharmacological activities, respectively. During the process of ripening from green to red tomatoes, the rate of conversion of tomatine to esculeoside A was high in the green tomatoes with a low tomatine content, whereas green tomatoes with a high tomatine content exhibited a low conversion rate. Using methanol extracts prepared from unripe and ripe fruits of the original cultivar and its mutant lines and two major compounds, we examined their cytotoxicity against FaDu human hypopharynx squamous carcinoma cells. Only tomatine exhibited cytotoxicity with an IC_50_ value of 5.589 μM, whereas the other samples did not exhibit cytotoxicity. Therefore, radiation breeding represents a useful tool for developing new cultivars with high quality, and metabolite analysis is applicable for the rapid and objective selection of potential mutant lines.

## 1. Introduction

Tomato (*Solanum lycopersicum* L.; formerly *Lycopersicon esculentum* Mill.; Solanaceae) is the second most important vegetable crop after potato (*Solanum tuberosum* L.), with around 182.3 million tons of tomato fruit produced annually on 4.85 million hectares [1]. Tomato is a fruit containing a lot of health-promoting compounds and is widely consumed fresh and used in cooking. Therefore, several techniques are being used to breed excellent tomato varieties, focusing on improving tomato fruit quality as important as improving yield and stress tolerance [2].

Mature red tomatoes are desirable because of their flavor and high amount of lycopene, which is a carotenoid that exhibits antioxidant effects [3]. Steroidal glycosides have also been identified; spirosolane-type glycoalkaloids from the stems and leaves [4]; tomatine (**1**) as a bitter compound in the unripe green fruit and leaf [3,5], esculeoside A (**2**) abundant in the ripe red fruit [6,7], and a pregnane glycoside, 3β-hydroxy-5α-pregn-16-en-20-one 3-*O*-β-lycotetraoside, from the overripe fruit [4].

Tomatine content decreases during the ripening process, and it is completely converted to esculeosides and lycoperosides once the tomato fruit turns red [5,8]. The degradation of tomatine involves several steps, including the hydroxylation of α-tomatine to hydroxytomatine, the acetylation of hydroxytomatine to acetoxy-hydroxytomatine, and the glycosylation of acetoxy-hydroxytomatine to esculeoside A, mediated by several enzymes related in the metabolic conversion of α-tomatine to esculeoside A [9,10,11,12]. Other studies have demonstrated that biosynthesis of esculeoside A from α-tomatine is associated with the ripening hormone, ethylene [13,14,15]. Tomatine is a solanum alkaloid similar to solanine, which is a well-known toxic glycoalkaloid in potatoes [16]; however, in vivo studies have demonstrated that oral tomatine administration is less toxic or non-toxic compared with other glycoalkaloids [17]. Tomatine has also been reported to exhibit several biological activities including anticancer [18,19], immunostimulatory [20], anti-inflammatory [21], and antibiotic [22] effects. Esculeoside A was isolated and identified as the major constituent in ripe red tomatoes by Fujiwara et al. [6,7]. Esculeoside A also has various pharmacological effects including anti-atherosclerosis [23], anti-hyaluronidase [24,25], anti-arthritis [26], and hypoglycemic [27] activity.

As part of our efforts to develop new varieties of *S. lycopersicum* cv. Micro-Tom with high functionality and improved quality, we generated mutant lines by radiation breeding with improved yields of phytochemicals. A comparative study of tomatine and esculeoside A content at two ripening stages of the original cultivar and the mutant lines was measured using ultra-performance liquid chromatography coupled with evaporative light scattering detector (UPLC-ELSD). In addition, the conversion rate of tomatine to esculeoside A was also determined at two ripening stages per line based on their concentrations in each sample.

## 2. Results and Discussion

### 2.1. Quantitative Analysis of Two Steroidal Glycoalkaloids in Micro-Tom Mutant Lines at Two Different Ripening Stages

**Figure 1:**
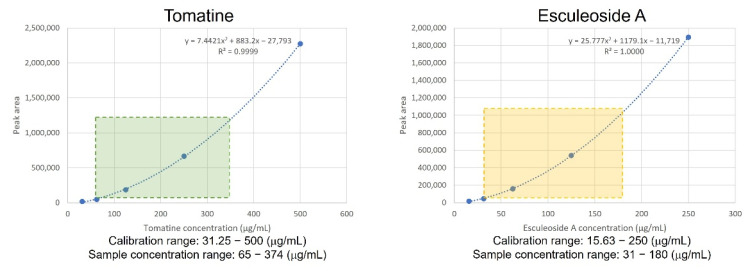
Polynomial-order 2 regression for two steroidal alkaloids identified from the micro-tomato samples. y and x are peak area and concentration of standard solution, respectively. *R*^2^ is coefficient value.

To establish steroidal glycoalkaloid profiles based on the fruit maturation period between the original cultivar (*S. lycopersicum* cv. Micro-Tom) and its mutant lines, UPLC-ELSD analysis was conducted with two standards, tomatine and esculeoside A, along with methanol extracts prepared from the tomatoes. Esculeoside A was isolated from methanol extracts of commercially available red micro-tomatoes and tomatine was obtained from the Sigma Chemical Co. (St. Louis, MO, USA). The structures were identified by analysis of the ^1^H and ^13^C NMR spectra and by comparing the data with published values [6]. Chromatographic conditions were optimized with regard to the separation of the peaks for the two compounds by UPLC-ELSD, which may be applied as a quick, universal, and convenient analytical method for the evaluation of these compounds, which are UV transparent. A major peak for tomatine and esculeoside A were observed in the chromatograms of the unripe green tomato samples and the ripe red tomato samples, respectively. For quantitation, calibration curves of tomatine and esculeoside A were prepared using five concentrations (tomatine: 31.25, 62.50, 125.00, 250.00, and 500.00 μg/mL; esculeoside A: 15.63, 31.25, 62.50, 125.00, and 250.00 μg/mL). As shown Table 1, the polynomial-order 2 equations for tomatine and esculeoside A were y = 7.4421x^2^ + 883.2x − 27,793 and y = 25.777x^2^ + 1179.1x − 11,719, respectively. The calibration curves were curvilinear with high correlation coefficients (*R*^2^ = 0.999 − 1.000) (Figure 1). This analytical method was used to measure tomatine and esculeoside A levels in the unripe green and ripe red fruits of the original cultivar and its mutant lines.

To select target-specific mutant lines, we screened the mutants for high or low relative tomatine content in the immature fruit and high esculeoside A content in the mature fruit. The peak area values were measured by ELSD for the original cultivar and 184 mutant lines at two different ripening stages (Figure S1). Tomatine has a dual aspect with anti-nutritive properties due to toxicity, along with potential for health promotion [5]. Therefore, selection of a mutant line with reduced toxicity due to a low level of tomatin accumulation and a mutant line with a high level of tomatine accumulation as a source of tomatine showing various pharmacological activities are proposed. To develop tomato cultivars with less toxicity and less bitterness targeting green immature fruits, the top 10 mutant lines with low tomatine content among the immature fruits were selected and designated 14-3, 14-5, 15-4, 18-7, 19-2, 20-5, 22-6, 26-2, 41-1, and 44-5. There have been reports of the consumption of an edible tomato cultivar (cherry tomato, *L. esculentum* var. *cerasiforme*) with a very high tomatine content (0.5–5 mg/g of dry weight) and in recipes, such as pickled or fried green tomatoes [28]. Tomatine has also been reported to exhibit various pharmacological activities. For example, tomatine inhibits the growth of human ovarian (Skov3) and breast (MCF-7) cancer cells [18,19]. It also shows immunostimulating effects as a vaccine adjuvant against infectious diseases and cancer immunotherapy [20]. Tomatine exhibits anti-inflammatory effects by suppressing NF-κB and JNK signaling in lipopolysaccharide-stimulated mouse macrophages [21] and it exerts antibiotic activities against microorganisms [22]. Thus, to develop tomato cultivars having value as an important source for extracting tomatin with various pharmacological activities, we selected the top 10 mutant lines with a higher content of tomatine compared with the original cultivar, which were designated 13-8, 15-8, 20-7, 26-8, 27-1, 27-7, 37-4, 42-3, 42-5, and 43-2. Esculeoside A is abundant in mature tomatoes and exhibits various biological activities. For example, it was shown to inhibit atherogenesis in apoE-depicient mice [23]. Esculeoside A also inhibited hyaluronidase activity and ameliorated dermatitis in 2,4-dinitro-chlorobenzen-treated mice [24]. Tomato saponin, including esculeoside A ameliorated collagen-induced arthritis in vivo through Th1 inhibition and Th2 activation [24], and it exhibited a hypoglycemic effect in db/db mice through the AMP-activated protein kinase/insulin receptor substrate-1 pathway [27]. Thus, the top 10 mutant lines with the highest esculeoside A content among the mature fruits were selected and designated 13-6, 13-8, 16-7, 17-3, 17-8, 18-2, 22-1, 22-6, 23-1, and 25-2.

To quantify tomatine and esculeoside A in the target-specific mutant lines, an established extraction and analytical method was used. The content of these two compounds in the mutant lines (μg/g of fresh weight) are presented in Table 1, Table 2 and Table 3. Table 1 shows the content of the two triterpenoid glycosides in unripe green fruits for the selected mutant lines with low tomatine content. The lowest accumulation of tomatine was observed in the mutant line 44-5 with a concentration of 299.1 ± 3.7 μg/g, which was approximately 83.22% lower compared with the wild type (1782.9 ± 7.2 μg/mL). When the same mutant line matured into red fruit, the mutant line 44-5 exhibited an esculeoside A quantity of 444.0 ± 3.2 μg/g, indicating that the conversion rate from tomatine to esculeoside A for the mutant line 44-5 was the highest at 122.16%. Therefore, this mutant line was considered a robust source of tomatine to esculeoside A. Table 2 shows the content of the two compounds in unripe green fruits for the selected mutant lines a high tomatine content. The mutant line 42-3 exhibited the highest accumulation of tomatine with a concentration of 1894.8 ± 7.9 μg/g, which represented 6.29% increase compared with the original cultivar. In the mature red fruit of the mutant line 42-3, the esculeoside A content was 281.7 ± 2.5 μg/g and its conversion rate from tomatine to esculeoside A was very low at 22.62%. This indicates an opposite tendency with respect to the conversion rate of the mutant lines with low tomatine content. Among these mutant lines, the ripe red fruit of the mutant line 37-4 contained tomatine and esculeoside A levels of 510.2 ± 5.5 μg/g and 428.6 ± 2.3 μg/g, respectively. For this mutant line, it was assumed that the metabolic conversion from tomatine to esculeoside A was not an efficient process. Table 3 lists the content of the two compounds in ripe red fruits for the selected mutant lines with high esculeoside A content. The highest value for esculeoside A was observed in line 13-6 at 786.0 ± 4.2 μg/g of fresh weight, which was 196% higher compared with that of the original cultivar (399.8 μg/g of fresh weight). This indicates a positive impact of the irradiation on improving the production of esculeoside A. This was more evident when comparing the quantitation results for esculeoside A content in the fresh ripe tomatoes in the market, which ranged from 92.8 to 456 μg/g of fresh weight [29]. Therefore, this mutant line is a promising candidate as a highly functional cultivar.

**Figure 2:**
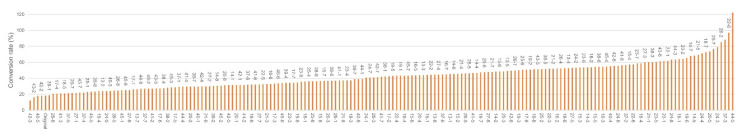
Conversion rate (%, ■) from tomatine to esculeoside A in each tomato sample.

We found that the high content of tomatine in immature fruits almost disappeared when the fruits ripened. Instead, the esculeoside A content increased in mature fruits, which was consistent with other studies [6]. According to a recent study on the metabolic pathway of steroid glycosides, glycosylation of tomatidine to tomatin requires four glycosyltransferases: GAME1, GAME17, GAME18 and GAME2 [8]. Additionally, the catalytic action of GAME31 (also known as Sl23DOX) leads to the hydroxylation of tomatine, and GAME5 is involved in the production of esculoside A [9,10,11,12]. The metabolic pathways for steroidal glycosides based on ripening characteristics have been also suggested [13,14,15]. For example, the high level accumulation of tomatine and the absence of esculeoside A were confirmed in tomato fruit tissues of non-ripening and ripening-inhibitor mutants [13]. It has been demonstrated that there is a glycosylation step in the biosynthesis pathway from tomatine to esculeoside A, which is dependent upon ethylene treatment during fruit ripening [14]. The hydroxylation at C-27 of the terpenoid backbone of tomatine result in C22βN→C22αN isomerization, which leads to the biosynthesis of esculeoside A, was proposed by quantum chemical calculations [15]. In addition, the content of various triterpenoid glycosides other than tomatine and esculeoside A in HPLC-ELSD-MS profiles of extracts from several tomato varieties has also been reported, and the total amount in tomato fruit remains somewhat constant during ripening [15]. Based on these studies, we compared the conversion rate of tomatine to esculeoside A in each micro-tomato mutant line (Figure 2). For the mutant lines with a low tomatine content, the conversion rate of tomatine to esculeoside A tended to be high as the fruit maturated, whereas the mutant lines with high tomatine content exhibited a low conversion rate from tomatine to esculeoside A. Mutant lines containing tomatine at the ripening stage were also found: 13-2, 13-5, 17-7, 22-4, 23-3, 24-5, 24-7, 26-1, 26-5, 27-4, 37-4, 39-5, 40-8, 41-2 and 45-2 (15 out of 184). The quantitative results for these mutant lines are shown in Table 4.

### 2.2. Cytotoxic Activities

**Figure 3:**
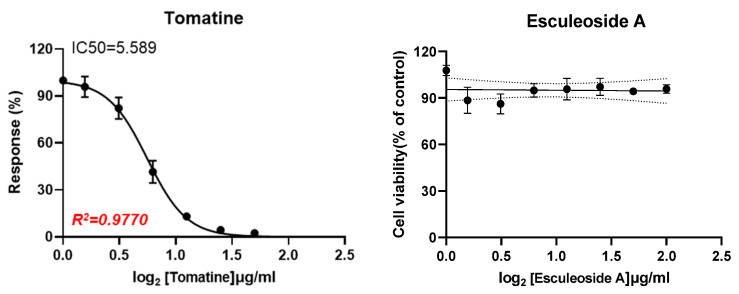
Cytotoxic effects of tomatine and esculeoside A in a dose-dependent manner on the Fadu cell line. Data represent the mean ± SD (n = 3).

Tomato steroidal glycosides have exhibited anti-proliferative activities against various cancer cell lines, including breast MCF-7, melanoma B16F2F, colon CT-26, liver HepG2, prostatic PC3, and lung A549 cancer cell lines [30,31]. However, the anticancer activities of micro-tomato, tomatine, and esculeoside A have not been examined in head and neck squamous cell carcinoma (HNSCC) [32]. Therefore, we measured the cytotoxic activity of methanol extracts from unripe and ripe fruits of the original micro-tomato cultivar and its mutant lines, tomatine, and esculeoside A against FaDu cells. Treatment with the methanol extract of the unripe green fruit and ripe red fruit of the original micro-tomato cultivar resulted in 85.89% ± 5.19% and 85.96% ± 4.97% cell viability at 50 μg/mL, respectively, indicating that their cytotoxic effect was weak (Tables S1 and S2). There were micro-tomato mutant lines that showed lower cell viability compared with that of the original cultivar. Of these, a value of 70.94% ± 2.56% was obtained for the unripe green fruit (14-4) and 70.18% ± 1.77% for the ripe red fruit (18-1) (Tables S1 and S2). Thus, the cytotoxicity of micro-tomato extracts against the FaDu cell line was considered insignificant. However, tomatine exhibited an IC_50_ value of 5.589 μM, whereas esculeoside A did not show any activity (Figure 3). Although this finding did not indicate a correlation between the content of tomatine in the extracts of micro-tomato samples and the cytotoxicities of the extracts of the micro-tomato samples, a potential anti-tumor effect of tomatine in FaDu cells may be expected and further studies regarding its mechanism of action and activity in vivo are needed.

## 3. Materials and Methods

### 3.1. General Procedures

A [^60^Co]-irradiator (150 TBq capacity; AECL, Ottawa, ON, Canada) at the Advanced Radiation Technology Institute (Jeongeup-si, Korea) was used to generate gamma radiation. A proton linear accelerator (TR103) at the Korea Multi-purpose Accelerator Complex (Gyeongju, Korea) was used for proton beam-irradiation. The nuclear magnetic resonance (NMR) experiments were carried out on a 600 MHz Bruker AVANCE NEO NMR spectrometer (Oxford magnet, Bruker Switzerland AG, Fällanden, Switzerland) operated with Bruker TopSpin software (Billerica, MA, USA) at the Core Research Support Center for Natural Products and Medical Materials (CRCNM). An ultra-performance liquid chromatography-evaporative light scattering detector (UPLC-ELSD) was used along with a Waters Acquity UPLC system (Waters, Milford, MA, USA) and an Alltech Model 3300 ELSD detector (BÜCHI, Flawil, Switzerland) equipped with a Kinetex C18 column (2.1 mm × 10 mm, 2.6 mm; Phenomenex, Torrance, CA, USA). A highly porous polystyrene gel (Diaion HP-20; Sigma Chemical Co., St Louis, MO, USA), reverse phase C18 (YMC gel ODS-A, 12 nm, S-75 μm, YMC Co., Kyoto, Japan) and Sephadex LH-20 (Cytiva, MA, USA) resin was used for column chromatography (CC). Thin-layer chromatography was performed on a Kieselgel 60 RP-18-F254S device (Merck, Darmstadt, Germany) with visualization under UV light (254 and 365 nm) and by heating at 180 °C for 2 min after spraying with 10% (*v/v*) sulfuric acid. The standard compound, tomatine, was obtained from Sigma-Aldrich (Sigma Chemical Co., St Louis, MO, USA). All of the other chemicals and solvents used in this study were of analytical grade.

### 3.2. Plant Materials

The mutant tomato fruit lines were generated by irradiating tomato seeds (*S. lycopersicum* cv. Micro-Tom) with gamma-rays [LET = 0.2 keV/μm] at 350 Gy for mutant lines 13–22, using 100 MeV proton beam [LET = 0.7306 keV/μm] at 100 Gy for mutant lines 23–27, a 100 MeV proton beam at 150 Gy for mutant lines 38–40, and 100 MeV proton beam at 200 Gy for mutant lines 41–45. All mutant lines and the original cultivar were M_3_ populations obtained after two cycles of cultivation and were grown in a greenhouse under the same conditions. Three fruits were randomly harvested from the M_3_ individuals for each mutant line three months after seeding (June and July 2020). The collected fresh fruits were frozen at −80 °C until further analysis. Voucher specimens (lines 13–45) were deposited at the Advanced Radiation Technology Institute, Korea Atomic Energy Research Institute.

### 3.3. Isolation and Identification of Esculeoside A from the Ripe Red Micro-Tomato

Fresh fruits (510 g) from commercially available red micro-tomato (*S. lycopersicum*) were homogenized in water using a mixer and the homogenate was centrifuged and filtered through Whatman No. 2 filter paper. The filtrate was passed through a Diaion HP-20 sequentially by eluting with water and methanol. The methanol eluate was then subjected to reverse phase C18 CC and eluted with a gradient solvent system (40% to 60% methanol in water) to yield 12 fractions (F01–F12). F09 was subjected to an open column packed with sephadex LH-20 using methanol as an eluent. This resulted in the isolation of esculeoside A (**2**, 15.4 mg).

Tomatine (**1**): White powder. LR-ESIMS (positive ions) *m*/*z* 1034.4 [M + H]^+^.

Esculeoside A (**2**): White powder. ^1^H (600 MHz, pyridine-*d*_5_) and ^13^C (150 MHz, pyridine-*d*_5_) NMR spectra, see Figures S2 and S3. LR-ESIMS (positive ions) *m*/*z* 1270.6 [M + H]^+^.

### 3.4. Preparation of Standard Solutions and Sample Preparation

Fresh unripe or ripe tomato fruits (the mutant lines and its original cultivar) were individually weighed and ground using a Geno/grinder (SPEX, New York, NJ, USA). The ground sample was mixed with methanol at a ratio of 10 mL methanol per 10 g and then extracted by sonication for 1 h. The extracted solutions were centrifuged (12,000× *g* for 10 min at 20 °C) and the supernatants were filtered through a polyvinylidene fluoride (PVDF) syringe filter (0.45 μm). The filtrates were then freeze-dried. Each of the methanol extracts was accurately weighed and dissolved in methanol at 6 mg/mL and then filtered through a PVDF syringe filter (0.45 μm) for HPLC analysis. Each of the standards, tomatine and esculeoside A, were accurately weighed and dissolved in methanol and 50% methanol in water, respectively, at 1.0 mg/mL. The stock solutions were diluted to prepare a series of standard solutions at five concentrations (tomatine: 31.25, 62.50, 125.00, 250.00, and 500.00 μg/mL; esculeoside A: 15.63, 31.25, 62.50, 125.00, and 250.00 μg/mL) for quantitative analysis.

### 3.5. Quantitative Analysis

Quantitative analyses were performed using a Waters Acquity UPLC system with a Kinetex C18 column. An Alltech Model 3300 ELSD detector was used for analyte detection. The temperature of the drift tube and the flow rate of the carrier gas (nitrogen) were set at 1.5 L/min and 40 °C, respectively. All of the data were processed using Empower 2 software (Waters, Milford, MA, USA). The mobile phase consisted of water containing 0.05% formic acid and 5% acetonitrile (*v*/*v*; solvent A) and 0.1% formic acid in acetonitrile (*v*/*v*; solvent B), and was run as follows: 0–10 min, 5–52.5% B; 10–11 min, 52.5–100% B; 11–13 min, 100% B; 13–13.01 min, 100–5%; and 13.01–15 min, 5% B. The flow rate was maintained at 0.3 mL/min and the injection volume was 2 μL. Each experiment was conducted in triplicate and all of the data are presented as the mean ± standard deviation (SD).

### 3.6. Cytotoxicity Assay

FaDu human pharynx squamous carcinoma cells were purchased from the Korean Cell Line Bank (Seoul, Korea) and cultured in a Dulbecco’s Modified Eagle’s Medium (Hyclone, Logan, UT, USA) supplemented with 10% fetal bovine serum (Gibco, Grand Island, NY, USA) in a humidified incubator. The viability of the cells was determined using a crystal violet assay [33]. Briefly, FaDu cells were seeded into 96-well plates at a density of 5 × 10^3^ cells/well and incubated at 37 °C for 24 h. Cultured FaDu cells were treated with the indicated concentration of extract (100 μg/mL), tomatine (1.5–100 μg/mL), and esculeoside (1.5–100 μg/mL). After a 48 h incubation, the cells were washed twice with PBS and stained with 0.5% crystal violet (in 20% methanol) for 20 min. The FaDu cells were washed with water 4 times and dried overnight. Thereafter, 200 μL of methanol was added and incubated for 20 min at room temperature. The optical density (OD) was measured at 584 nm using an ELISA reader (Softmax Pro., Molecular Devices, San Jose, CA, USA). The percentage of cell viability was calculated using the following formula:

Cell viability (%) = Test OD/Control OD × 100

The 50% inhibitory concentration (IC_50_) values were calculated from a dose–response analysis performed using GraphPad Prism software (GraphPad Software, La Jolla, CA, USA).

## 4. Conclusions

The content of two major steroidal alkaloids in the unripe green and ripe red fruits of *S. lycopersicum* cv. Micro-Tom, and its mutant lines were analyzed by UPLC-ELSD. Selection of mutant lines with a low content of tomatine may induce the development of green tomatoes, which may increase consumption due to reduced toxicity and less bitterness. Esculeoside A abundant in red tomatoes, contains 4 to 5 times more than lycopene, a powerful antioxidant, and has medicinal effects in preventing arteriosclerosis and regulating blood pressure. Thus, selection of mutant lines with a high content of esculeoside A may lead the development of new red tomato cultivars with high functionality and improved quality. Furthermore, it can be used as industrial materials for the development of cardiovascular-related health functional foods or drugs. In addition, the extracts of the tomato samples and the two compounds were evaluated for cytotoxicity in FaDu cells for the first time in this study. Tomatine exhibited potent cytotoxicity, thus it may represent a lead compound with anticancer effects in HNSCC cancer cells and further studies on its mechanism of action are warranted.

## Figures and Tables

**Table 1:** Content of two triterpenoid glycosides (μg/g of fresh weight) in the top 10 mutant lines with low tomatine content among the immature green fruits of the mutant tomato lines.

Mutant Lines	Ripening Stage	Tomatine	Esculeoside A
14-3	immature green	961.4 ± 5.9	nd
	mature red	nd	296.4 ± 3.2
14-5	immature green	502.9 ± 1.7	nd
	mature red	nd	356.1 ± 3.7
15-4	immature green	660.0 ± 7.8	nd
	mature red	nd	356.4 ± 2.0
18-7	immature green	508.9 ± 3.3	nd
	mature red	nd	348.9 ± 3.9
19-2	immature green	782.0 ± 9.0	nd
	mature red	nd	298.8 ± 3.3
20-5	immature green	452.8 ± 2.7	nd
	mature red	nd ^2^	414.5 ± 7.9
22-6	immature green	428.8 ± 6.2	nd
	mature red	nd	495.7 ± 0.3
26-2	immature green	346.1 ± 2.6	nd
	mature red	nd	204.0 ± 1.8
41-1	immature green	575.5 ± 8.1	nd
	mature red	nd ^2^	231.7 ± 2.0
44-5	immature green	299.1 ± 3.7 ^1^	nd ^2^
	mature red	nd	444.0 ± 3.2
Original cultivar	immature green	1782.9 ± 7.2	nd
	mature red	nd	399.8 ± 1.3

^1^ Values are mean ± standard deviation in triplicate. ^2^ nd: not detected.

**Table 2:** Content of two triterpenoid glycosides (μg/g of fresh weight) in the mutant lines with a higher tomatine content compared with the original cultivar among unripe green fruits of mutant tomato lines.

Mutant Lines	Ripening Stage	Tomatine	Esculeoside A
13-8	immature green	1494.6 ± 17.6	nd
	mature red	nd	698.9 ± 4.6
15-8	immature green	1430.8 ± 10.1	nd
	mature red	nd	597.5 ± 4.6
20-7	immature green	1537.3 ± 15.9	nd
	mature red	nd	366.7 ± 1.4
26-8	immature green	1299.2 ± 11.0	nd
	mature red	nd	377.6 ± 2.7
27-1	immature green	1198.0 ± 4.0	nd
	mature red	nd	281.0 ± 4.0
27-7	immature green	1266.6 ± 13.3	nd
	mature red	nd	546.3 ± 4.6
37-4	immature green	1700.8 ± 14.3	nd
	mature red	510.2 ± 5.5	428.6 ± 2.3
42-3	immature green	1894.8 ± 7.9 ^1^	nd ^2^
	mature red	nd	281.7 ± 2.5
42-5	immature green	1392.4 ± 8.5	nd
	mature red	nd	560.4 ± 8.9
43-2	immature green	1702.8 ± 4.0 ^1^	nd
	mature red	nd	283.6 ± 4.0
Original cultivar	immature green	1782.9 ± 7.2	nd
	mature red	nd	399.8 ± 1.3

^1^ Values are mean ± standard deviation in tinplate. ^2^ nd: not detected.

**Table 3:** Content of two triterpenoid glycosides (μg/g of fresh weight) in the top 10 mutant lines with high esculeoside A content among the ripe red fruits of mutant tomato lines.

Mutant Lines	Ripening Stage	Tomatine	Esculeoside A
13-6	immature green	1429.4 ± 7.4	nd
	mature red	nd	786.0 ± 4.2
13-8	immature green	1494.6 ± 17.6 ^1^	nd ^2^
	mature red	nd	698.9 ± 4.6
16-7	immature green	1044.5 ± 10.8	nd
	mature red	nd	555.6 ± 3.2
17-3	immature green	1153.2 ± 14.8	nd
	mature red	nd	447.5 ± 4.8
17-8	immature green	1156.6 ± 8.6	nd
	mature red	nd	676.3 ± 1.8
18-2	immature green	1178.6 ± 13.1	nd
	mature red	n	479.7 ± 3.1
22-1	immature green	900.1 ± 15.8	nd
	mature red	nd	556.1 ± 2.9
22-6	immature green	428.8 ± 6.2	nd
	mature red	nd	495.7 ± 0.3
23-1	immature green	947.7 ± 5.4	nd
	mature red	nd	697.6 ± 3.7
25-2	immature green	993.2 ± 11.0	nd
	mature red	nd	620.4 ± 1.3
Original cultivar	immature green	1782.9 ± 7.2	nd
	mature red	nd	399.8 ± 1.3

^1^ Values are mean ± standard deviation in triplicate. ^2^ nd: not detected.

**Table 4:** Content of two triterpenoid glycosides (μg/g of fresh weight) in the mutant lines with tomatine content at the ripening stage.

Mutant Lines	Ripening Stage	Tomatine	Esculeoside A
13-2	immature green	1103.5 ± 8.2 ^1^	nd ^2^
	mature red	413.8 ± 3.6	371.4 ± 4.0
13-5	immature green	975.7 ± 6.7	nd
	mature red	510.1 ± 6.2	550.1 ± 1.9
17-7	immature green	946.5 ± 9.7	nd
	mature red	335.3 ± 2.8	433.2 ± 1.6
22-4	immature green	817.7 ± 20.6	nd
	mature red	236.9 ± 7.9	413.9 ± 6.7
23-3	immature green	855.8 ± 6.9	nd
	mature red	555.5 ± 8.2	317.5 ± 7.0
24-5	immature green	983.2 ± 17.6	nd
	mature red	344.2 ± 2.5	317.7 ± 4.1
24-7	immature green	1093.2 ± 6.4	nd
	mature red	419.4 ± 4.2	549.7 ± 2.5
26-1	immature green	971.5 ± 10.0	nd
	mature red	259.8 ± 4.1	392.1 ± 3.9
26-5	immature green	1025.6 ± 15.4	nd
	mature red	207.7 ± 1.3	499.6 ± 4.1
27-4	immature green	1072.1 ± 24.6	nd
	mature red	278.0 ± 4.2	512.0 ± 4.0
37-4	immature green	1700.8 ± 14.3	nd
	mature red	510.2 ± 5.5	428.6 ± 2.3
39-5	immature green	1173.3 ± 14.6	nd
	mature red	322.3 ± 0.2	360.5 ± 3.1
40-8	immature green	909.5 ± 20.6	nd
	mature red	332.9 ± 1.2	417.2 ± 3.3
41-2	immature green	1274.6 ± 22.5	nd
	mature red	622.8 ± 4.3	409.7 ± 1.0
45-2	immature green	781.5 ± 11.1	nd
	mature red	258.0 ± 1.0	300.3 ± 2.6
Original cultivar	immature green	1782.9 ± 7.2	nd
	mature red	nd	399.8 ± 1.3

^1^ Values are mean ± standard deviation in triplicate. ^2^ nd: not detected.

## Data Availability

Data are contained within the article and Supplementary Material.

## Supplementary Materials

The following are available online at https://www.mdpi.com/article/10.3390/plants11212895/s1, Figure S1: The screen for relative content of (**a**) tomatine (■) in unripe fruits and (**b**) esculeoside A (■) and tomatine (■) in ripe fruits of the original cultivar of micro-tomato and its mutant lines, Figure S2: ^1^H NMR spectrum of esculeoside A (600 MHz, pyridine-*d*_5_), Figure S3: ^13^C NMR spectrum of esculeoside A (150 MHz, pyridine-*d*_5_), Table S1: Cell viabilities (%) of the methanol extract of unripe green fruit of the micro-tomato original cultivar and mutant lines (n = 4), Table S2: Cell viabilities (%) of the methanol extract of ripe red fruit of the micro-tomato original cultivar and mutant lines (n = 4).
