# Supplementary material for: Content of Two Major Steroidal Glycoalkaloids in Tomato (Solanum lycopersicum cv. Micro-Tom) Mutant Lines at Different Ripening Stages
Source: Plants (Basel). 2022 Oct 28;11(21):2895. doi: 10.3390/plants11212895 (PMC9654965; doi:10.3390/plants11212895)
Supplement: Supplementary file 1 [file plants-11-02895-s001.zip › plants-1950645-supplementary.pdf]

Supplementary Materials

Content of Two Major Steroidal Glycoalkaloids in Tomato (*Solanum lycopersicum* cv. Micro-Tom) Mutant Lines at Different Ripening Stages

Trung Huy Ngo [1,†], Jisu Park [2,†], Yeong Deuk Jo [2,3], Chang Hyun Jin [2], Chan-Hun Jung [4], Bomi Nam [5], Ah-Reum Han [2,*] and Joo-Won Nam [1,*]

[1] College of Pharmacy, Yeungnam University, Gyeongsan-si 38541, Gyeongsangbuk-do, Korea
[2] Advanced Radiation Technology Institute, Korea Atomic Energy Research Institute, Jeongeup-si 56212, Jeollabuk-do, Korea
[3] College of Agriculture and Life Sciences, Chungnam National University, Daejeon 34134, Chungcheongnam-do, Korea
[4] Jeonju AgroBio-Materials Institute, Jeonju-si 54810, Jeollabuk-do, Korea
[5] Institute of Natural Cosmetic Industry for Namwon, Namwon-si 55801, Jeollabuk-do, Korea
* Correspondence: arhan@kaeri.re.kr (A.-R.H.); jwnam@yu.ac.kr (J.-W.N.); Tel.: +82-63-570-3167 (A.-R.H.); +82-53-810-2818 (J.-W.N.)
† These authors contributed equally to this work.

Contents

Figure S1. The screen for relative content of (**a**) tomatine (■) in unripe fruits and (**b**) esculeoside A (■) and tomatine (■) in ripe fruits of the original cultivar of micro-tomato and its mutant lines.

Figure S2. ^{1}H NMR spectrum of esculeoside A (600 MHz, pyridine-d_5).

Figure S3. ^{13}C NMR spectrum of esculeoside A (150 MHz, pyridine-d_5).

Table S1. Cell viabilities (%) of the methanol extract of unripe green fruit of the micro-tomato original cultivar and mutant lines (n=4).

Table S2. Cell viabilities (%) of the methanol extract of ripe red fruit of the micro-tomato original cultivar and mutant lines (n=4).

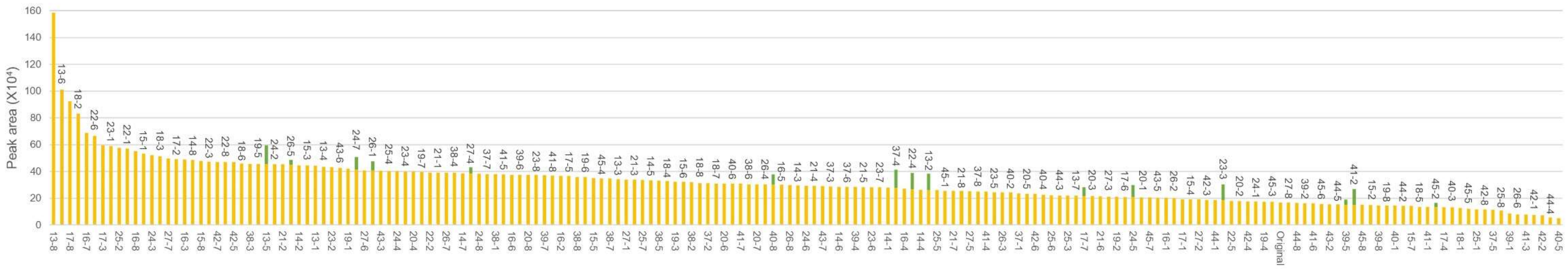

(**a**)

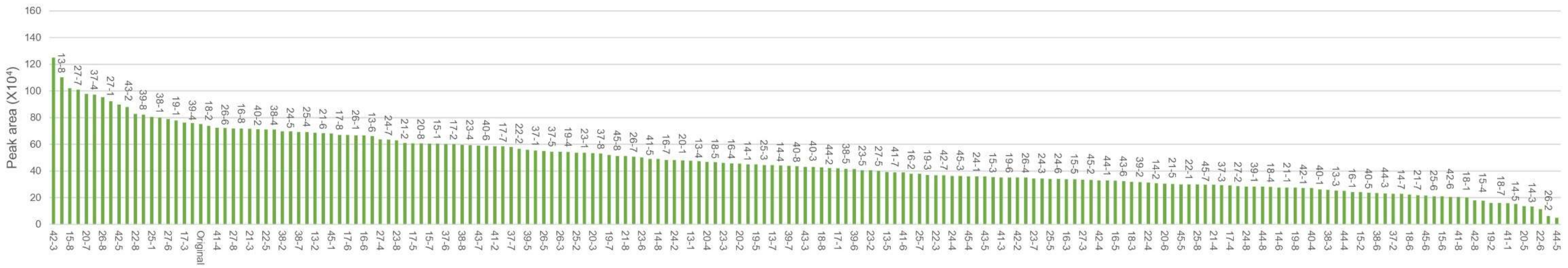

(**b**)

Figure S1. The screen for relative content of (**a**) tomatine (■) in unripe fruits and (**b**) esculeoside A (■) and tomatine (■) in ripe fruits of the original cultivar of micro-tomato and its mutant lines.

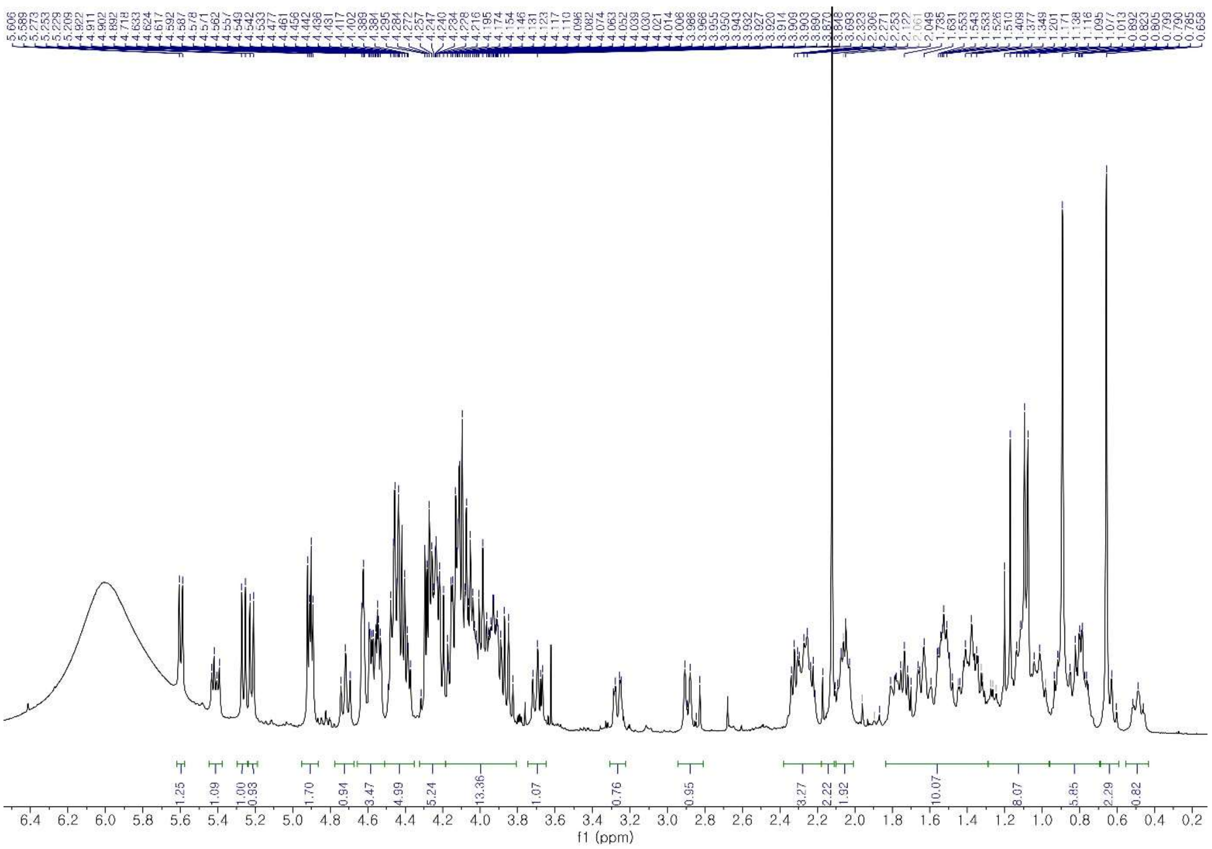

Figure S2. ^{1}H NMR spectrum of esculeoside A (600 MHz, pyridine-d_5).

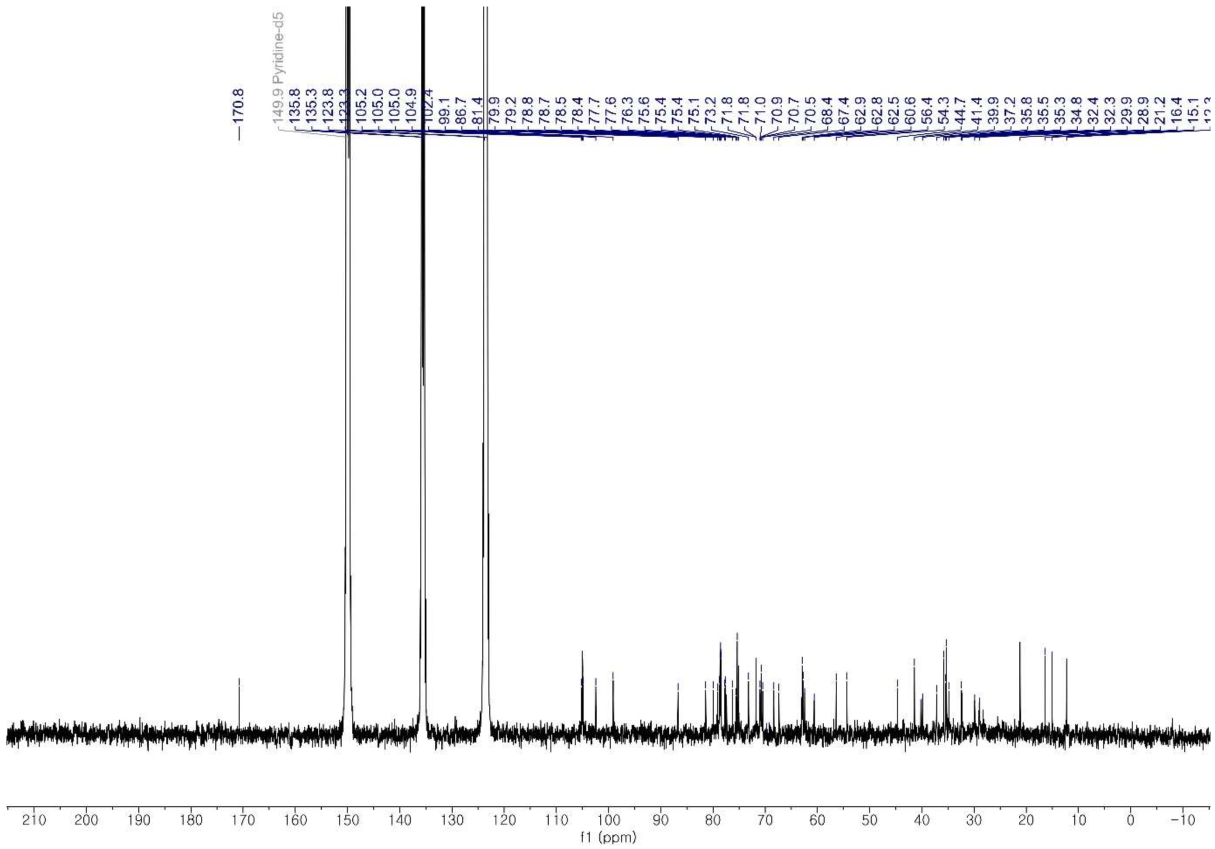

Figure S3. ^{13}C NMR spectrum of esculeoside A (150 MHz, pyridine-d_5).

Table S1. Cell viabilities (%) of the methanol extract of unripe green fruit of the micro-tomato original cultivar and mutant lines (n=4).

sample	mean	SD
vehicle	100.00	5.45
cisplastin	46.03	3.74
original-G	85.89	5.19
13-1	91.39	9.66
13-2	81.96	9.75
13-3	91.75	6.94
13-4	89.68	8.03
13-5	93.19	2.73
13-6	91.14	6.82
13-7	102.02	1.94
13-8	80.07	2.14
14-1	82.78	4.56
14-2	79.88	5.64
14-3	86.24	3.78
14-4	70.94	2.56
14-5	94.74	3.73
14-6	92.06	2.07
14-7	96.99	6.23
14-8	81.06	2.42
15-1	77.21	4.34
15-2	76.97	2.36
15-3	82.58	1.77
15-4	82.66	4.09
15-5	91.34	3.56
15-6	79.18	2.43
15-7	89.93	6.40
15-8	85.56	4.54
16-1	88.75	3.49
16-2	84.84	2.90
16-3	86.48	3.58
16-4	90.86	7.19

sample	mean	SD
16-5	87.87	4.22
16-6	87.00	9.00
16-7	91.76	5.10
16-8	90.40	0.34
17-1	87.82	5.72
17-2	89.15	9.20
17-3	82.24	9.24
17-4	84.03	12.09
17-5	93.19	5.51
17-6	85.44	2.86
17-7	95.42	13.10
17-8	84.85	3.38
18-1	85.05	5.53
18-2	82.88	4.04
18-3	86.39	7.00
18-4	86.35	5.42
18-5	93.67	9.78
18-6	85.42	0.54
18-7	88.56	2.33
18-8	84.56	3.22
19-1	72.83	2.96
19-2	80.01	0.10
19-3	81.28	1.59
19-4	92.23	0.90
19-5	91.29	15.37
19-6	88.30	10.47
19-7	82.89	2.46
19-8	84.47	3.45
20-1	82.08	3.93
20-2	76.80	2.31
20-3	83.93	3.43

sample	mean	SD
20-4	91.45	7.10
20-5	96.63	5.34
20-6	87.01	12.77
20-7	88.03	6.37
20-8	83.56	18.10
21-1	89.15	23.33
21-2	89.23	14.56
21-3	92.12	3.79
21-4	86.69	6.37
21-5	90.37	5.01
21-6	93.21	11.23
21-7	97.77	6.70
21-8	91.71	2.96
22-1	88.03	5.63
22-2	98.08	14.01
22-3	85.99	4.01
22-4	84.42	3.16
22-5	85.29	7.19
22-6	86.50	2.01
22-8	87.45	0.82
23-1	86.23	4.62
23-2	81.54	4.17
23-3	91.05	6.66
23-4	90.54	13.62
23-5	85.07	7.75
23-6	86.97	9.82
23-7	92.32	2.81
23-8	88.34	8.23
24-1	83.18	3.13
24-2	90.87	14.04
24-3	93.55	11.43

sample	mean	SD
24-4	97.23	15.39
24-5	94.09	13.40
24-6	96.03	11.15
24-7	99.43	12.29
24-8	97.97	9.10
25-1	100.56	8.25
25-2	101.14	6.71
25-3	93.01	5.86
25-4	103.21	4.51
25-5	109.87	9.34
25-6	102.30	3.00
25-7	117.43	3.56
25-8	103.96	15.31
26-1	108.72	8.68
26-2	107.66	7.34
26-3	90.19	4.09
26-4	92.86	3.23
26-5	93.10	6.04
26-6	102.94	13.62
26-7	100.91	22.35
26-8	95.67	10.71
27-1	103.16	10.30
27-2	111.69	3.86
27-3	96.49	9.71
27-4	101.38	4.02
27-5	84.94	13.07
27-6	103.16	8.64
27-7	98.12	9.19
27-8	102.58	10.91
37-1	107.76	5.92
37-2	90.09	16.63

sample	mean	SD
37-3	80.47	4.64
37-4	94.25	2.43
37-5	106.91	4.52
37-6	94.13	3.45
37-7	85.03	1.98
37-8	84.00	3.96
38-1	77.59	4.19
38-2	85.63	7.52
38-3	81.14	0.97
38-4	89.34	9.73
38-5	78.32	4.97
38-6	74.37	1.54
38-7	85.14	8.31
38-8	94.02	2.10
39-1	80.03	4.81
39-2	81.30	2.24
39-4	87.40	5.30
39-5	86.10	4.59
39-6	81.74	5.87
39-7	89.81	9.20
39-8	89.69	3.99
40-1	91.35	11.33
40-2	88.57	12.82
40-3	87.23	3.15
40-4	85.42	5.15
40-5	85.01	4.68
40-6	85.33	5.53
40-8	77.65	14.55
41-1	97.59	3.71
41-2	97.41	7.12
41-3	99.93	6.86

sample	mean	SD
41-4	107.31	0.65
41-5	106.77	5.38
41-6	102.61	2.86
41-7	98.45	2.54
41-8	98.43	5.57
42-1	94.47	3.35
42-2	93.94	4.02
42-3	94.28	1.09
42-4	97.61	7.25
42-5	100.87	3.40
42-6	96.64	5.14
42-7	104.31	8.75
42-8	99.84	3.99
43-2	99.60	3.49
43-3	95.85	1.25
43-5	99.95	7.03
43-6	95.53	2.41
43-7	96.50	1.59
44-1	101.41	5.15
44-2	106.03	14.97
44-3	101.02	8.62
44-4	100.73	4.41
44-5	105.28	1.36
44-8	103.21	3.19
45-1	98.47	3.70
45-2	102.51	1.96
45-3	99.70	4.16
45-4	89.64	9.37
45-5	90.34	1.96
45-6	91.78	3.75
45-7	89.82	10.30
45-8	79.14	9.06

Table S2. Cell viabilities (%) of the methanol extract of ripe red fruit of the micro-tomato original cultivar and mutant lines (n=4).

sample	mean	SD
vehicle	100.00	3.55
cisplastin	43.47	5.23
original-R	85.96	4.97
13-1	97.52	5.70
13-2	83.92	10.18
13-3	92.44	4.57
13-4	92.40	0.91
13-5	101.56	5.13
13-6	101.07	7.36
13-7	100.94	3.48
13-8	88.46	5.54
14-1	99.10	2.37
14-2	102.11	2.30
14-3	101.55	2.28
14-5	102.61	5.07
14-6	107.01	3.05
14-7	107.81	6.92
14-8	102.46	5.83
15-1	101.40	3.31
15-2	98.29	5.73
15-3	101.67	3.37
15-4	106.48	2.10
15-5	107.21	4.15
15-6	101.55	0.25
15-7	106.89	3.65
15-8	104.66	4.58
16-1	104.66	3.90
16-2	97.43	6.85
16-3	90.52	7.10
16-4	97.74	3.12
16-5	75.37	3.49
16-6	80.90	3.23
16-7	81.32	8.07
16-8	85.49	14.30
17-1	100.89	3.60
17-2	95.55	0.91
17-3	100.12	4.45
17-4	93.85	6.62
17-5	79.72	6.78
17-6	93.24	2.12
17-7	83.28	8.07
17-8	86.81	6.68
18-1	70.18	1.77
18-2	97.72	3.54
18-3	99.45	2.49
18-4	97.45	10.97
18-5	97.19	5.75
18-6	97.40	3.39
18-7	97.56	4.46
18-8	90.67	17.09
19-1	91.42	7.98
19-2	96.35	13.91
19-3	95.38	3.98
19-4	92.63	4.89
19-5	93.33	5.23
19-6	94.17	14.02
19-7	89.43	14.92
19-8	103.19	4.01
20-1	101.58	7.46
20-2	92.11	2.12
20-3	95.98	5.19
20-4	88.88	2.21

sample	mean	SD
20-5	100.18	1.58
20-6	98.24	5.80
20-7	95.82	9.41
20-8	105.15	6.71
21-1	103.06	5.03
21-2	90.34	1.54
21-3	97.68	5.89
21-4	104.91	5.43
21-5	91.02	14.75
21-6	95.10	3.16
21-7	103.17	3.47
21-8	102.03	3.23
22-1	89.88	9.24
22-2	93.77	2.89
22-3	94.23	10.81
22-4	95.03	5.31
22-5	79.47	3.27
22-6	89.71	7.06
22-8	103.79	3.57
23-1	96.54	9.46
23-2	97.56	4.56
23-3	92.90	0.30
23-4	89.00	4.78
23-5	85.62	2.89
23-6	92.54	8.20
23-7	93.89	6.16
23-8	93.72	8.83
24-1	99.70	4.73
24-2	98.45	4.96
24-3	96.10	0.81
24-4	83.86	0.71

sample	mean	SD
24-5	92.29	6.39
24-6	87.36	3.59
24-7	89.35	5.40
24-8	91.69	7.90
25-1	103.01	13.09
25-2	90.87	2.28
25-3	95.09	3.69
25-4	83.03	0.62
25-5	94.14	3.18
25-6	101.85	5.85
25-7	91.46	11.22
25-8	102.87	2.48
26-1	94.30	1.96
26-2	95.45	3.22
26-3	100.25	9.53
26-4	97.74	4.06
26-5	97.66	6.91
26-6	104.04	10.80
26-7	102.60	7.37
26-8	104.16	2.96
27-1	89.16	1.91
27-2	91.92	7.60
27-3	92.07	3.15
27-4	94.17	4.76
27-5	96.88	1.97
27-6	98.01	9.13
27-7	98.38	2.41
27-8	86.93	3.33
37-1	88.00	7.07
37-2	95.63	3.75
37-4	94.99	2.96

sample	mean	SD
37-5	86.84	1.06
37-6	91.21	6.10
37-7	88.67	1.84
37-8	92.99	3.89
38-1	90.95	5.80
38-2	88.80	1.19
38-3	87.01	0.39
38-4	88.76	1.79
38-5	86.69	2.42
38-6	92.42	6.79
38-7	90.05	7.32
38-8	87.35	6.58
39-1	90.91	3.40
39-2	87.54	3.44
39-3	92.37	1.48
39-4	94.12	1.18
39-5	89.47	3.95
39-6	100.75	3.98
39-7	127.14	20.87
39-8	104.45	5.43
40-1	97.77	2.47
40-2	105.70	4.38
40-3	108.47	4.08
40-4	109.92	4.39
40-5	109.08	3.19
40-6	100.81	1.90
40-8	103.49	3.62
41-1	108.99	1.75
41-2	106.97	3.73
41-3	102.24	2.62
41-4	103.70	4.40

sample	mean	SD
41-5	109.71	3.12
41-6	103.11	6.28
41-7	92.63	7.41
41-8	96.95	7.17
42-1	94.32	11.36
42-2	94.96	9.87
42-3	92.78	13.65
42-4	88.97	9.27
42-5	100.21	7.48
42-6	97.26	10.00
42-7	89.11	10.06
42-8	102.08	4.05
43-2	90.94	1.71
43-3	85.58	4.14
43-5	86.78	12.32
43-6	87.23	7.62
43-7	102.98	1.86
44-1	95.00	2.53
44-2	91.23	4.18
44-3	91.96	5.68
44-4	89.23	7.22
44-5	96.03	5.48
44-8	90.49	6.93
45-1	94.50	6.73
45-2	105.01	2.20
45-3	96.57	5.22
45-4	93.35	4.33
45-5	90.42	9.12
45-6	93.80	1.02
45-7	88.28	2.31
45-8	89.39	1.67
